# Anesthetic Management of Patients With Anterior Mediastinal Masses Undergoing Chamberlain Procedure (Anterior Mediastinostomy)

**DOI:** 10.5812/ircmj.2120

**Published:** 2013-04-05

**Authors:** Alireza Sharifian Attar, Reza Jalaeian Taghaddomi, Reza Bagheri

**Affiliations:** 1 Department of Anesthesia, Chaem Hospital, Mashhad University of Medical Sciences, Mashhad, IR Iran; 2 Department of Tthoracic Surgery, Ghaem Hospital, Mashhad University of Medical Sciences, Mashhad, IR Iran

**Keywords:** Mediastinoscopy, Laryngeal Masks, Propofol

*Dear Editor,*

Patients with mediastinal masses, particularly masses in the anterior or superior mediastinum present unique problems for the anesthesiologists which are during general anesthesia, which are usually the consequence of extrinsic compression of the airway, obstruction of the venous return or obstruction to the output of the heart (1-4).

The aim of our study was to describe the anesthetic management of patients who underwent anterior mediastinostomy to biopsy masses or lymph nodes in the center of the chest (Chamberlain Procedure). 50 consecutive patients scheduled for Chamberlain Procedure were enrolled in the study. Patients with severe orthopnea, morbid obesity and concomitant asthma were excluded. Complications were classified as mild (abnormality noted but no significant change in practice required e.g. mild elevation in measured ETCo2), moderate (abnormality noted requiring change in practice or additional therapy e.g. moderate airway obstruction responding to change in patient position) or severe (abnormality requiring rapid intervention to avert potentially dangerous deterioration e.g. airway obstruction requiring intubation or passage of a rigid bronchoscope) (5).

Anesthetic management: After 3 minutes of preoxygenation, in all patients, anesthesia was induced with midazolam (1-2mg), fentanyl ( 1-1.5μg/kg), propofol (1.5-2mg/kg) and it was maintained with continuous infusion of propofol( 50μg/kg/min) and sufentanil (0.01 μg/kg/min). An appropriate size laryngeal mask airway (LMA) was inserted for all patients, and assisted mechanical ventilation was accomplished. Routine monitoring including ECG, pulse oximetry, NIBP, ETCo2 and monitoring of airway pressure were applied to all patients. The main anesthetic challenge of this procedure is intraoperative collapse of the tracheobronchial tree which may lead to severe hypoxia and even death. 10 Patients had no respiratory or cardiovascular problem before operation. Table 1 shows the respiratory symptoms and signs in 40 patients.

**Table 1.** Patients’ Number With Clinical Signs at Presentation

Clinical Sign	Male No. (%)	Female No. (%)
**None**	3 (6)	7 (14)
**Cough**	20 (40)	18 (36)
**Dyspnea**	12 (24)	11 (22)
**Orthopnea**	21 (42)	19 (38)
**Stridor**	8 (16)	4 (8)
**Wheeze**	5 (10)	3 (6)
**SVC obstruction**	12 (24)	12 (24)

CT Scan of the patients revealed CT Tracheobronchial diameter < 50% in 31 patients and it was more than 50% in other patients. There were no perioperative deaths. Intraoperative complications were noted in 6 patients. Only 2 of the complications could be classified as cardiovascular in origin: one a transient fall in blood pressure, which corrected by fluid infusion, and the other one, arrhythmias as bigiminal PVC which treated by lidocaine (60gm). The remaining 4 cases were classified as respiratory in origin: 3 cases were graded mild or moderate, requiring either no or limited intervention. The remaining one found airway collapse which ventilation was impossible. The airway obstruction was relieved by rigid bronchoscopy and lateral position. In this case, the operation continued ad the patient was awake at the end of the surgery.

During induction of general anesthesia in patients with an anterior or superior mediastinal masses, airway obstruction is the most common and feared complication. It is important to note that the point of tracheobronchial compression usually occurs distal to an ETT. Management of these patients is guided by their symptoms and the CT scan. Patients with uncertain airways should have diagnostic procedures performed under local or regional anesthesia whenever possible. Patients with uncertain airways requiring general anesthesia need a step-by-step induction of anesthesia with continuous monitoring of gas exchange and hemodynamics. This “NPIC” (noli pontes ignii consumere, i.e, “don’t burn your bridges”) anesthetic induction can be an inhalation induction with a volatile agent or intravenous titration of propofol with or without ketamine, maintaining spontaneous ventilation until either the airway is definitively secured or the procedure is completed.

Awake intubation of the trachea before induction is a possibility in some adult patients if the CT scan shows an area of noncompressed distal trachea to which the endotracheal tube can be advanced before induction. If muscle relaxants are required, ventilation should first be gradually taken over manually to ensure that positive-pressure ventilation is possible and only then can a short-acting muscle relaxant be administered. In our study, all patients were anesthetized using intravenous anesthetics to maintain spontaneous ventilation and counteracting the mechanisms which facilitate tumor compression of the airway, muscle relaxants were avoided. This has been highly recommended in the anesthetic management of anterior mediastinal masses. To avoid manipulation of the patients, airway and endotracheal intubation and also because of the proximity of the surgical field to patients’ airway and difficulty with mask ventilation, we used LMA to keep the patients’ airway open. In our study, only one patient needed a change in position and rigid bronchoscopy to alleviate airway obstruction intraoperatively, while Ferrari and Bedford5 reported two patients who required airway management with a rigid bronchoscope, two patients who needed a change in position to alleviate airway obstruction, and four patients who could not be extubated at the end of surgery. We concluded that in Chamberlain Procedure, this method of anesthesia means intravenous anesthesia without muscle relaxant and using LMA to manage the airway, is a relatively safe method of anesthesia. In this method, the patients are not awake and seem to be more comfortable. So, not only the spontaneous ventilation is maintained, but also we have a relatively secure airway.
